# Associations between Accelerometer-Measured Physical Activity and Fecal Microbiota in Adults with Overweight and Obesity

**DOI:** 10.1249/MSS.0000000000003096

**Published:** 2022-12-22

**Authors:** RILEY L. HUGHES, DOMINIKA M. PINDUS, NAIMAN A. KHAN, NICHOLAS A. BURD, HANNAH D. HOLSCHER

**Affiliations:** 1 Department of Food Science and Human Nutrition, University of Illinois at Urbana–Champaign, Urbana, IL; 2 Department of Kinesiology and Community Health, University of Illinois at Urbana–Champaign, Urbana, IL; 3 Beckman Institute for Advanced Science and Technology, University of Illinois at Urbana–Champaign, Urbana, IL; 4 Neuroscience Program, University of Illinois at Urbana–Champaign, Urbana, IL; 5 Division of Nutrition Sciences, University of Illinois at Urbana–Champaign, Urbana, IL

**Keywords:** GUT MICROBIOTA, SHORT-CHAIN FATTY ACIDS, ACCELEROMETERS, SEDENTARY TIME

## Abstract

**Purpose:**

We aimed to assess whether total daily physical activity (PA), PA intensities, sedentary time (ST), and prolonged ST are associated with differences in the gut microbiota composition or short-chain fatty acid (SCFA) profile of adults with overweight or obesity.

**Methods:**

Cross-sectional associations between total daily PA (counts per minute), PA intensities (light and moderate-to-vigorous (MVPA)), ST, prolonged ST, and fecal microbiota composition were assessed in adults (*n* = 124) between 25 and 45 yr of age with body mass index ≥25 kg·m^−2^. Fecal microbiota composition was assessed with 16S rRNA gene sequencing. Daily PA and ST were measured with a hip-worn ActiGraph wGT3X-BT accelerometer.

**Results:**

Daily PA volume and intensity were positively associated with relative abundance of *Faecalibacterium* (*P* = 0.04) and negatively associated with the abundances of *Alistipes*, *Parabacteroides*, and *Gemmiger* (*P* = 0.003–0.04) as well as the concentrations of acetate, butyrate, and total SCFA (all *P* = 0.04). Conversely, ST was negatively associated with abundance of *Faecalibacterium* but positively associated with the abundances of taxa, including Ruminococcaceae, *Parabacteroides*, *Alistipes*, and *Gemmiger*. Clustering of participants based on whether they met PA recommendations suggested that SCFA profiles differed between individuals who did and did not meet PA recommendations. K-means clustering based on percent of time spent in MVPA and ST also identified differences in fecal microbiota composition between cluster 1 (lower MVPA, higher ST) and cluster 2 (higher MVPA, lower ST), including a higher abundance of *Alistipes* in cluster 1.

**Conclusions:**

The current analysis suggests a beneficial association of daily PA on the fecal microbiota and a negative association of ST, particularly with respect to the associations of these variables with the genera *Faecalibacterium*, a butyrate-producing taxon.

Physical activity (PA) is defined as any body movement produced by skeletal muscles that requires energy expenditure (1,2). Engagement in any PA intensity reduces the risk of premature mortality and a myriad of chronic diseases (1,3,4) and promotes physical and mental health (3). When the intensity of PA is considered, higher moderate-to-vigorous PA (MVPA) of any duration can benefit cardiometabolic and cardiovascular health and decrease the risk of premature mortality (5). However, growing evidence suggests that light-intensity PA is also associated with a reduced risk of premature mortality (4) and could help improve cardiometabolic health (6). Conversely, physical inactivity (i.e., noncompliance with PA guidelines of ≥150 min of moderate, >75 min of vigorous, or any equivalent combination of the two intensities per week) (7) was identified as the fourth leading risk factor for global mortality by the World Health Organization (8) and has a higher prevalence than all other risk factors (9). Sedentary time (ST) is associated with greater mortality as well as gastrointestinal inflammation (i.e., increased circulating lipopolysaccharide) and noncommunicable diseases including colorectal cancer, cardiovascular disease, and diabetes, independent of recreational PA (10,11). ST is not the same as physical inactivity or lack of exercise, as individuals who engage in regular PA and exercise may also have high levels of ST (12). Less than 10% of Americans meet the recommended amount of weekly MVPA based on accelerometry (13). Importantly, PA is a modifiable lifestyle factor, and evidence suggests that even small increases in daily PA can provide health benefits (3,4,9). However, the mechanisms by which PA prevents disease and improves health are not fully understood (1). In addition, there remains a large degree of interindividual variability in response to regular PA and exercise (i.e., planned, structured, and repetitive PA with the goal to maintain or improve fitness) (2,14). Much of this variability remains unexplained, although genetic factors are thought to contribute as well as compensatory metabolic and behavioral changes in response to increases in daily PA (14,15). Recent discussions have concluded that “-omics” data, including the gut microbiota, may help elucidate mechanisms underlying connections between daily PA and health and variability in the health effects of daily PA (1).

The effects of the gut microbiota extend far beyond the gastrointestinal system, influencing systemic functions including metabolism and immunity (16). These effects are mediated in part by the production of metabolites (17) (e.g., short-chain fatty acids (SCFA) and branched-chain fatty acids (BCFA)) that influence host systems and metabolic pathways (16). Variability in the composition of the gut microbiome (18) has fueled research on the relationship between features of the gut microbiota, such as diversity or the presence, absence, or the amount of certain taxa, and host health. In addition, the gut microbiota is not a fixed trait but instead responds to environmental stimuli and is a malleable part of the human supraorganism (19,20).

Diet is a lifestyle factor that directly influences the gut microbiota by providing substrates for microbial metabolism (21). In contrast, PA may influence the gut microbiota via more indirect mechanisms such as alterations in substrate utilization, gut transit time, bile acid profile, and signaling pathways in muscle and immune cells (19). Most of the research on PA and the gut microbiota has focused on aerobic-based exercise (19), a subcategory of PA, and has often focused on athletic populations. These studies have suggested that exercise influences the gut microbiota composition, often increasing the abundance of health-associated bacteria such as *Lactobacillus*, *Bifidobacterium*, and *Akkermansia* as well as increasing SCFA production and the abundance of butyrate-producing taxa (19). Large, cohort studies that have collected gut microbiota samples and metadata, including self-reported PA or exercise data, have not yet reported associations between the gut microbiota and PA (22,23). Few studies have investigated the relationship between objectively measured, total daily PA and PA intensity and the gut microbiota, particularly in young to middle-age adults (24).

The objective of this cross-sectional analysis is to assess whether total daily PA and PA intensities or ST and prolonged ST are associated with differences in the gut microbiota composition or SCFA profile of adults with overweight or obesity. We hypothesized that greater total daily PA and less ST would be associated with greater relative abundance of health-associated bacteria such as *Bifidobacterium*, *Lactobacillus*, and *Akkermansia* and SCFA- and butyrate-producing microbes such as *Faecalibacterium* as well as correspondingly greater fecal SCFA concentrations. Characterizing relations between total daily PA and PA intensities, ST, and prolonged ST and the fecal microbiota will help narrow the gap in understanding how daily PA contributes to human health.

## METHODS

### Study design

This cross-sectional analysis was performed on previously collected baseline data (before randomization/intervention) from the *Persea americana for Total Health* study (25). Adults between 25 and 45 yr of age with a body mass index (BMI; kg·m^−2^) ≥25.0 were enrolled in this study. Study exclusion criteria included the following: 1) BMI <25.0 kg·m^−2^, 2) pregnancy or lactating, 3) current tobacco use, 4) previous diagnosis of metabolic or gastrointestinal disease, 5) food allergies or intolerances, 6) use of medications that impact normal bowel function, or 7) malabsorptive or restrictive bariatric surgery within the previous 2 yr (25). Study procedures were administered in accordance with the Declaration of Helsinki and were approved by the University of Illinois Institutional Review Board. This trial is registered at www.ClinicalTrials.gov as NCT02740439.

Baseline dietary intake was assessed using the Dietary History Questionnaire II, a standardized and validated tool for nutritional assessment developed by the National Cancer Institute (26,27). Baseline dietary fiber intake (in grams per 1000 kcal) using the US Department of Agriculture values from the Dietary History Questionnaire was used in the linear analyses, as described hereinafter. Healthy Eating Index (HEI) total score was also compared between clusters, as described hereinafter.

### Fecal microbiota and metabolites

Participants collected fecal samples on their own and delivered the samples within 15 min of defecation as previously described (25). Briefly, upon arrival to the laboratory, samples were homogenized, placed in aliquots, flash frozen, and stored at −80°C for microbiota analysis. Fecal DNA was isolated, and the V4 region of the 16S ribosomal RNA gene was amplified then sequenced at the WM Keck Biotechnology Center as previously described (25). Sequences were demultiplexed in Quantitative Insights Into Microbial Ecology version 2 (QIIME2) version 2019.4, and amplicon sequence variants were generated using the DADA2 version 1.10.1 denoise-single plugin using default settings after dereplication and standard quality-filtering procedures (e.g., the removal of sequencing-related barcodes, sequences with quality scores <20, and chimeric sequences) (25). Taxonomy was assigned using the q2-feature-classifier command with default parameters in QIIME2, and sequences were matched against the Greengenes 13_8 database (25).

Fecal aliquots for volatile fatty acid analysis were weighed, acidified with 2 N HCl, and stored at −20°C until analysis. SCFA (butyrate, propionate, and acetate) and BCFA (isobutyrate, valerate, and isovalerate) concentrations were quantified using GC-LC (180 cm × 4 mm i.d. glass column with 10% SP-1200/1% HVFA H_3_PO_4_ on 80/100 mesh Chromosorb WAW; Hewlett-Packard 5890A Series II gas chromatograph; Supelco, Inc., Bellefonte, PA) and normalized on a dry matter basis (in micromoles per gram) (25).

### Accelerometry

A triaxial wGT3X-BT accelerometer (ActiGraph LLC., Pensacola, FL; 3.3 × 4.6 × 1.5 cm; 19 g; dynamic range, ±8*g*) was used to measure total daily PA, time spent in PA intensities, ST, and prolonged ST. The accelerometer was worn for 7 consecutive days on the right axillary line during waking hours, except for water-based activities. The raw accelerationsignal was sampled continuously at 100 Hz. Acceleration data were converted to vertical axis counts over 60-s epochs using ActiLife software (version 6.13.3; ActiGraph LLC., Pensacola, FL). Non–wear time was defined as 60 consecutive minutes of 0 counts (28) and excluded from the analyses. Only participants with at least 4 d with at least 10 h·d^−1^ of wear time were included in the analyses (29). Counts per minute was used as a measure of total daily PA (4). PA intensities were defined as follows: ≥100 to 2019 (light) and MVPA ≥2020 counts per minute, and expressed in minutes per day (29). ST was defined as <100 accelerometer counts per minute; prolonged ST was defined as time spent in sedentary bouts lasting ≥30 consecutive minutes (30) and not allowing for tolerance time ≥100 counts per minute (31). PA and ST variables are defined and described in Supplemental Table 1 (Supplemental Digital Content 1, http://links.lww.com/MSS/C763). Participants were classified according to adherence to the aerobic portion of PA recommendations (7). Specifically, those engaging in 150 min·wk^−1^ of MVPA-equivalent PA were classified as meeting PA recommendations. MVPA-equivalent PA was expressed as minutes of moderate PA plus twice the minutes spent in vigorous PA.

### Statistical analyses

Before analyses, all PA variables except for counts per minute (light PA, MVPA minutes per day), ST, and prolonged ST were adjusted for accelerometer wear time using the residuals method (32). Each variable was regressed on wear time, and unstandardized residuals were saved. The predicted value of the PA or ST variable was then computed using the mean wear time of the sample as a constant and added to the unstandardized residuals saved from the respective simple regression models for each PA and ST variable.

Associations between wear-time adjusted PA variables, ST, prolonged ST and fecal microbiota composition, and SCFA concentrations were first assessed using multivariate linear regression models. In R (version 4.0.4), phyloseq (version 1.34.0) was used to glom taxa at the phylum and genus levels. The top 5 phyla and top 20 genera were selected for analysis in the linear models. The Firmicutes-to-Bacteroidetes ratio and α-diversity (Faith’s PD and Shannon diversity) were also computed and used in the linear model analysis. Age, sex, BMI, and baseline dietary fiber intake were used as covariates in the model. Prolonged ST was further adjusted for total ST. Linearity assumptions were verified visually and sensitivity analysis for statistically significant outcomes with apparent outliers was performed to verify results.

Two strategies were used to group participants. First, participants were categorized on the basis of whether or not they met the recommendations for PA (7). K-means clustering was also used to categorize participants based on percent of time spent in MVPA and ST. Differences between clusters in age, sex, BMI, HEI total score, Firmicutes/Bacteroidetes (F/B) ratio, diversity indices, and SCFA were defined using Student’s *t*-tests or *χ*^2^ tests (for sex). Clusters were then analyzed to determine whether gut microbiota composition was significantly different between clusters using Analysis of Compositions of Microbiomes with Bias Correction (ANCOM-BC) (33) and DESeq2 (34). The formula used for the ANCOM-BC function included the same covariates as the linear models (i.e., age, sex, BMI, and baseline dietary fiber intake).

Results were considered significant if the *P* value was <0.05. Because of the exploratory nature of these analyses, results were not corrected for multiple hypothesis testing (35).

## RESULTS

### Participant characteristics

Of the 163 participants who underwent baseline testing, 124 participants had both microbiota and accelerometer data. The characteristics of these participants are shown in Table 1.

**TABLE 1:** Participant characteristics.

	Mean	Range
Age (yr), mean ± SD	34.9 ± 5.9	25–46
Sex		
Men (*n*)	44	
Women (*n*)	80	
Weight (kg), mean ± SD	94.5 ± 17.4	63.3–134.5
BMI (kg·m^−2^), mean ± SD	32.5 ± 5.4	25.0–48.0
PA and ST		
CPM	307.4 ± 106.5	106.5–617
Light (min·d^−1^)	259.2 ± 59.9	115.7–427.4
MVPA (min·d^−1^)	34.0 ± 19.1	1.2–95.8
ST (min·d^−1^)	615.3 ± 66.9	411.6–775.8
Prolonged ST (min·d^−1^)	196.0 ± 77.5	45.0–449.0

CPM, counts per minute.

### Linear model associations

**FIGURE 1:**
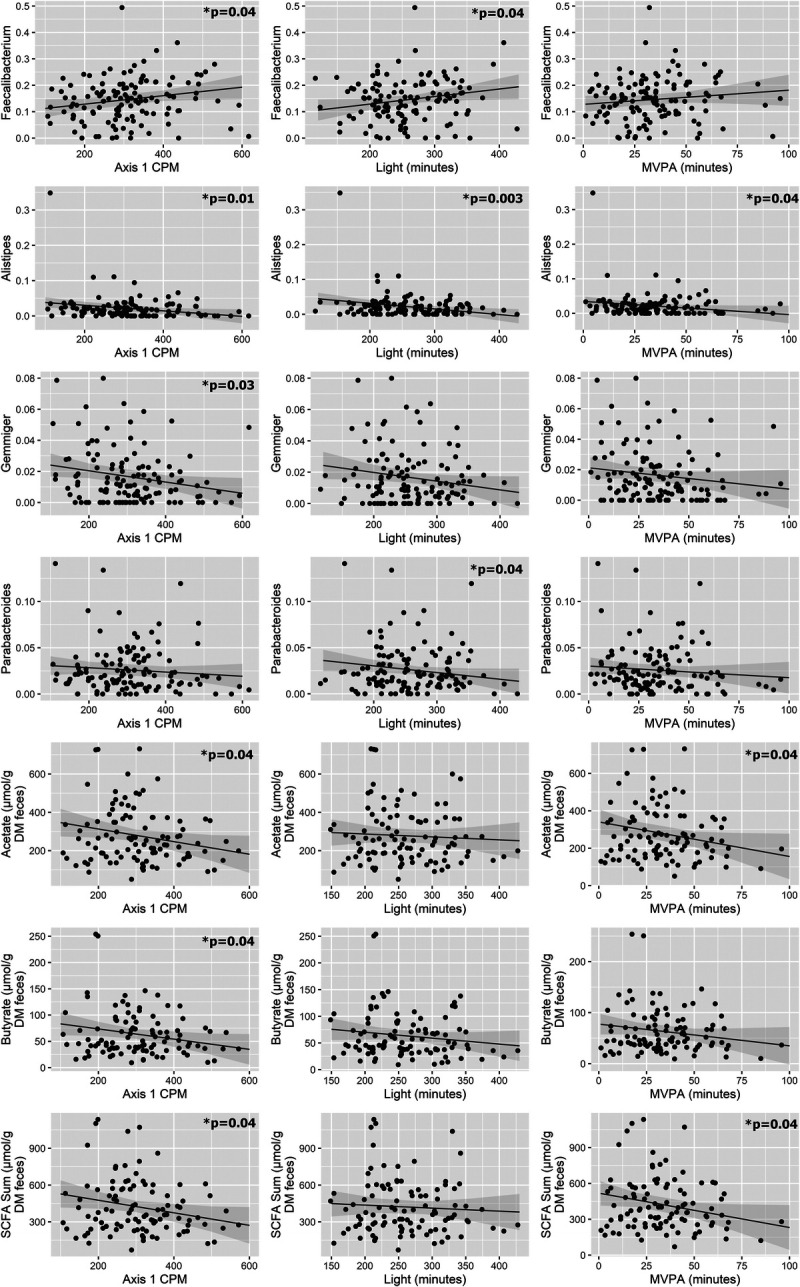
PA association with gut microbiota. Linear models controlled for age, sex, BMI, and baseline dietary fiber intake indicated significant associations between total PA and gut microbiota composition (proportions). CPM, counts per minute; DM, dry matter.

**FIGURE 2:**
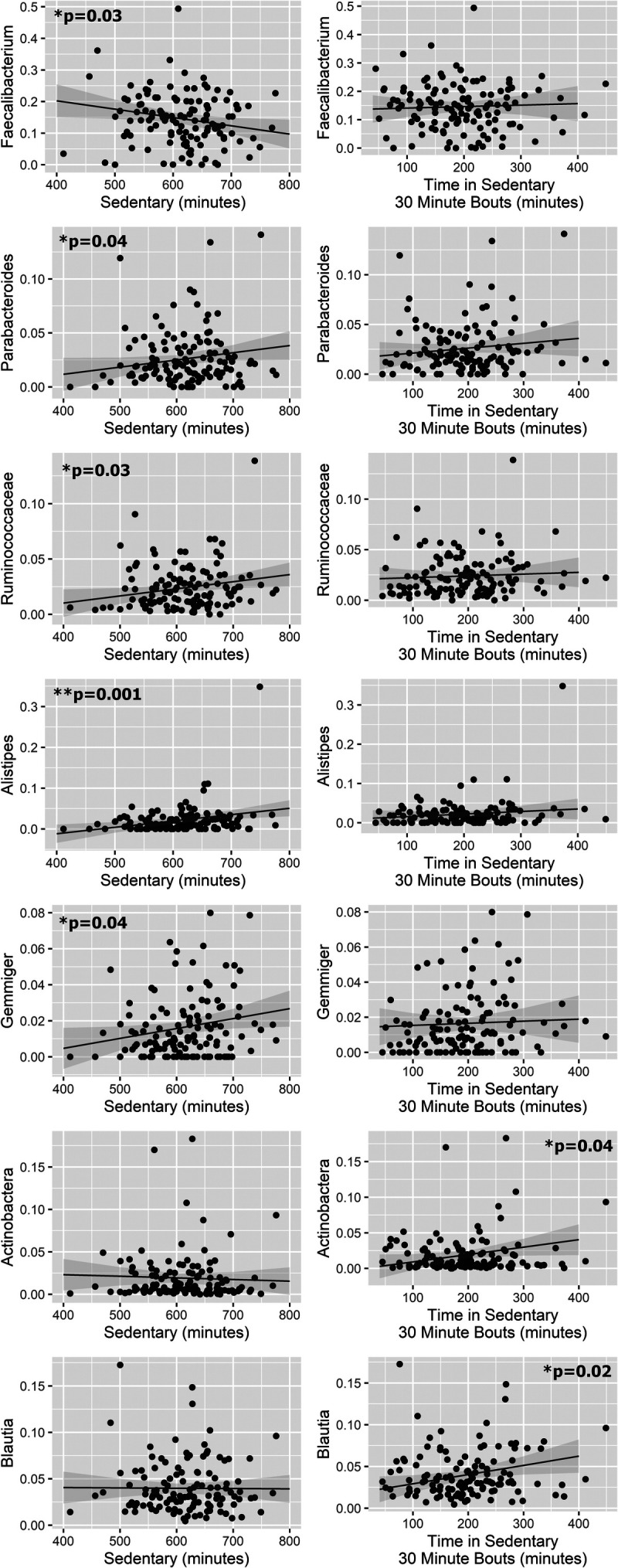
ST association with gut microbiota. Linear models controlled for age, sex, BMI, and baseline dietary fiber intake indicated significant associations between PA volume and gut microbiota composition (proportions).

The PA variables can be grouped into three categories: total daily PA (counts per minute), PA intensity (light, MVPA), and ST (ST and prolonged ST). Total daily PA and time spent in PA intensities were positively associated with relative abundance of *Faecalibacterium* (counts per minute, light PA) but negatively associated with relative abundance of *Alistipes* (counts per minute, light, MVPA), *Parabacteroides* (light), and *Gemmiger* (counts per minute) as well as concentrations of acetate (counts per minute, MVPA), butyrate (counts per minute), and total SCFA (counts per minute, MVPA; Fig. 1). However, after removal of gut microbiota and SCFA outliers via sensitivity analysis, there was only a trending relationship between *Alistipes* and counts per minute (*P* = 0.08), whereas the associations between *Alistipes* and MVPA (*P* = 0.17), and the association between butyrate and counts per minute (*P* = 0.33) were no longer statistically significant. Adjusting MVPA for ST revealed a negative association between MVPA and the SCFA/BCFA ratio (*P* = 0.03; Supplemental Table 2, Supplemental Digital Content 2, Linear model results, http://links.lww.com/MSS/C764). Conversely, ST was negatively associated with relative abundance of *Faecalibacterium* but positively associated with relative abundances of several taxa (Ruminococcaceae, *Parabacteroides*, *Alistipes*, and *Gemmiger*; Fig. 2). After adjusting ST for MVPA, only the association with *Alistipes* remained statistically significant (*P* = 0.01), although there was only a trending association after sensitivity analysis (*P* = 0.08; Supplemental Table 2, Supplemental Digital Content 2, Linear model results, http://links.lww.com/MSS/C764). Prolonged ST, adjusted for ST, was positively associated with Actinobacteria and *Blautia* (Fig. 2). These associations remained significant after also adjusting for MVPA (*P* = 0.03 and *P* = 0.03, respectively; Supplemental Table 2, Supplemental Digital Content 2, Linear model results, http://links.lww.com/MSS/C764).

### Clustering based on PA recommendations and K-means clustering

Clustering participants based on whether they complied with the aerobic portion of PA recommendations resulted in two clusters: compliers (*n* = 88) and noncompliers (*n* = 36). These two clusters did not differ in age, BMI, baseline dietary fiber intake, or HEI total score but were different in their sex distribution (*P* = 0.05; Supplemental Table 3, Supplemental Digital Content 3, Meet PA recommendations sex demographics, http://links.lww.com/MSS/C765).

The microbiota composition of these two clusters was also compared. F/B ratio and α-diversity were not different between the two groups, but there were statistically significant differences in SCFA profiles (Supplemental Fig. 1, Supplemental Digital Content 4, Differences in SCFA profiles based on meeting PA recommendations, http://links.lww.com/MSS/C766). Participants who did not meet weekly PA recommendations had lower concentrations of isovalerate and a higher SCFA/BCFA ratio compared with those who did meet recommendations. Comparison of the two groups using DESeq2 (Supplemental Fig. 2, Supplemental Digital Content 4, Meet PA cluster DESeq2 comparison, http://links.lww.com/MSS/C766, and Supplemental Table 4, Supplemental Digital Content 5, Meet PA recommendations DESeq2 results, http://links.lww.com/MSS/C767) also revealed differences in the relative abundances of several taxa, although analyses using ANCOM-BC did not reveal differences between groups (Supplemental Table 5, Supplemental Digital Content 6, Meet PA recommendations ANCOM-BC results, http://links.lww.com/MSS/C768).

**FIGURE 3:**
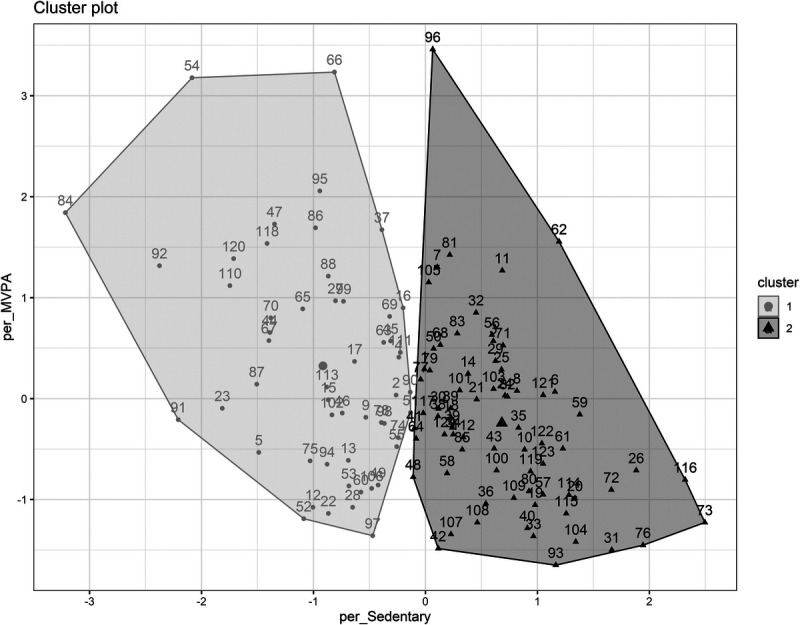
K-means clustering. K-means clustering based on percent of time spent in MVPA and ST with a k-value of two.

K-means clustering was used to group participants based on the percent of time spent in MVPA and ST, in accordance with previous research (30). Both elbow and silhouette methods were used to determine optimal number of clusters (36). A k-value of two was chosen, which resulted in cluster 1 (*n* = 71) and cluster 2 (*n* = 53) (Fig. 3).

Percent of time spent in MVPA and ST, age, sex, BMI, baseline fiber intake, HEI total score, SCFAs, F/B ratio, and α-diversity (Faith’s PD and Shannon diversity) were compared between the two clusters using *t*-tests or *χ*^2^ test for sex. There were no statistically significant differences in age, sex, BMI, HEI total score, SCFAs, F/B ratio, or α-diversity between the two clusters. Clusters differed in percent of time spent in MVPA and ST, with cluster 1 showing lower percent of time spent in MVPA (mean ± SD, 3.2% ± 1.9% vs 4.4% ± 2.3%) and higher percent of ST (mean ± SD, 72.8% ± 4.4% vs 60.9% ± 4.8%; Supplemental Fig. 3, Supplemental Digital Content 4, K-means cluster PA comparison, http://links.lww.com/MSS/C766).

**FIGURE 4:**
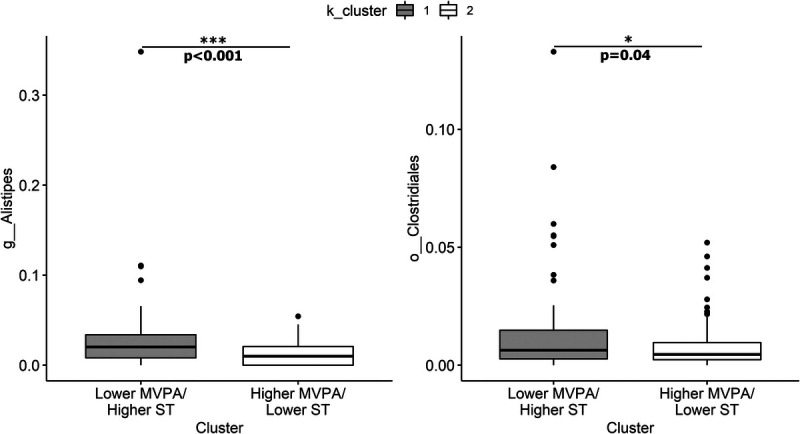
K-means cluster DESeq2 comparison. DESeq2 identified two taxa, *Alistipes* and Clostridiales, that were significantly different between clusters. Relative abundance of taxa displayed as proportions.

**FIGURE 5:**
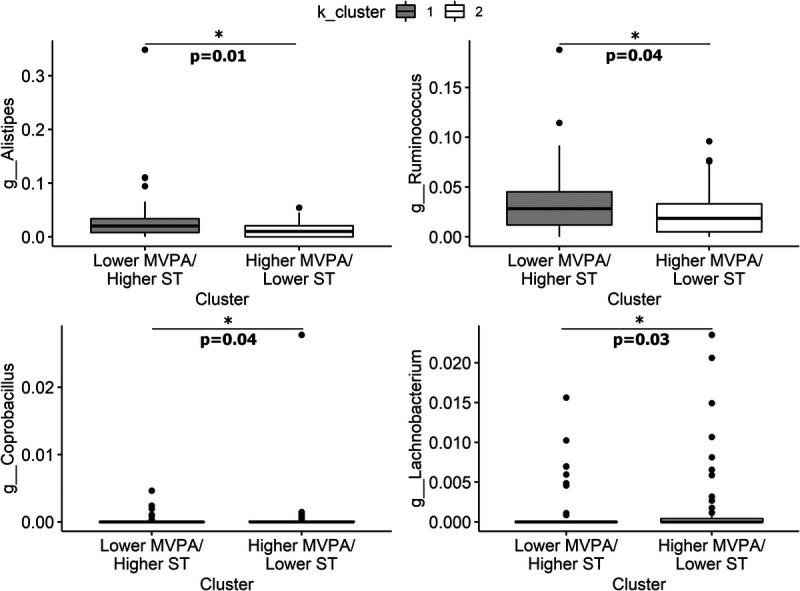
K-means cluster ANCOM-BC comparison. ANCOM-BC identified five taxa, *Alistipes*, *Ruminococcus*, *rc4–4*, *Coprobacillus*, and *Lachnobacterium*, that were significantly different between clusters. Relative abundance of taxa displayed as proportions.

ANCOM-BC and DESeq2 were used to compare the fecal microbial composition of the two clusters. DESeq2 identified two taxa, *Alistipes* and Clostridiales, that were significantly different between the two clusters, both showing higher relative abundance in cluster 1 (Fig. 4 and Supplemental Table 6, Supplemental Digital Content 7, K-means clustering DESeq2 results, http://links.lww.com/MSS/C769). Analysis with ANCOM-BC also revealed *Alistipes* as significantly different between clusters as well as *Ruminococcus*, *Coprobacillus*, and *Lachnobacterium* (Fig. 5 and Supplemental Table 7, Supplemental Digital Content 8, K-means clustering ANCOM-BC results, http://links.lww.com/MSS/C770).

## DISCUSSION

The current analysis is novel in that it is the only study, to our knowledge, that investigates the associations between gut microbiota composition and objectively measured PA in a population of adults with overweight and obesity. The results of our analyses indicate that there are associations between total daily PA, time spent in specific PA intensities (light PA and MVPA), and the fecal microbiota in this cohort of younger and middle-age adults with overweight and obesity.

The current analysis found associations between PA and ST and gut microbiota taxa that have been previously reported in associations between exercise or PA and the gut microbiota, although the directions of the associations in the current findings do not always match previous reports. For instance, ST showed positive associations with *Parabacteroides* and *Gemmiger*. These findings are in contrast with previous reports that these taxa were positively associated with onset of exercise in inactive older women (35) and juvenile rats (36), respectively. Comparison of individuals who did and did not meet weekly PA recommendations using DESeq2 also revealed differences in abundances of individual taxa such as *Ruminococcus* (family Lachnospiraceae), YS2, and *Haemophilus*, although ANCOM-BC revealed no significant results. Bressa et al. (24) reported *Haemophilus* to be increased in active women, which aligns with the results reported herein. According to the analyses using ANCOM-BC, *Lachnobacterium* and *Coprobacillus* relative abundances were higher in k-means cluster 2 (higher MVPA, lower ST). *Lachnobacterium* has previously been reported to be more abundant in individuals with low levels of PA (37), which is in contrast with the current analysis. *Coprobacillus* was decreased in athletes supplemented with *Lactobacillus plantarum* PS128 compared with a placebo group (38). This genus has also been associated with long-term intake of a Western-style diet (39).

The current analysis revealed associations between PA and SCFA-producing taxa as well as SCFA profiles. Linear models revealed that light PA was positively associated with relative abundance of *Faecalibacterium*. In contrast, ST showed a negative association with *Faecalibacterium*. The positive associations between counts per minute and light PA and *Faecalibacterium* are in agreement with previous studies investigating both total daily PA and exercise (24,40,41). *Faecalibacterium* is one of the most abundant butyrate-producing bacteria in the gastrointestinal tract and is proposed to have additional anti-inflammatory properties (42). Butyrate is an SCFA with beneficial health effects, including anti-inflammatory and immunomodulatory effects (42). Thus, the positive association between PA and this taxon has beneficial implications for human health, whereas the negative association between ST and this taxon could negatively impact gastrointestinal and metabolic health. Cluster analysis based on k-means clustering also revealed differences in SCFA-producing taxa. *Ruminococcus* (family Ruminococcaceae) was higher in cluster 1 (lower MVPA, higher ST) according to the analyses utilizing ANCOM-BC, reflecting the positive association between ST and Ruminococcaceae herein. This finding is also in agreement with results from Bressa et al. (24) that *Ruminococcus* was higher in sedentary versus active women. *Ruminococci* have been deemed key symbionts of the gut ecosystem because of their ability to metabolize complex polysaccharides and generate SCFA (43). However, SCFA has been reported to be elevated in adults with obesity in some studies (44), suggesting that SCFA-producing bacteria may also play a role in weight gain under certain circumstances by increasing energy harvest. The positive association between ST and Ruminococcaceae may reflect this association in the current cohort of adults with overweight and obesity. Previous research has shown that aerobic-based exercise has different effects on fecal SCFA concentrations in lean and obese individuals, increasing concentrations in lean individuals but not in individuals with obesity (45,46). This could explain the lack of positive association in the current cohort between total daily PA and fecal SCFA concentrations (acetate, butyrate, and total SCFA). Cluster analysis based on whether participants met weekly PA recommendations also revealed differences in SCFA profiles, including lower isovalerate concentrations and higher SCFA/BCFA ratio in those who did not meet the PA recommendations.

Lastly, the current analysis revealed associations between PA and taxa that have been implicated in interindividual variability in response to exercise. Our results of both the DESeq2 and ANCOM-BC analyses revealed that there was a higher abundance of *Alistipes* in k-means cluster 1 (lower MVPA, higher ST). This supports the finding from the linear models in the current analysis that total daily PA and light PA were negatively associated with relative abundance of *Alistipes*, and ST was positively associated with relative abundance of this taxon. This finding is also in agreement with previous studies that have reported a negative association between PA and *Alistipes* (47) or that a higher abundance of *Alistipes* was associated with greater ST or a nonathletic lifestyle (47,48). The role of the *Alistipes* genus in the gut ecosystem is not well understood (49). Some evidence suggests that *Alistipes* is associated with pathogenicity in colorectal cancer and depression, but other evidence suggests that it is protective against diseases such as liver fibrosis, colitis, cancer immunotherapy, and cardiovascular disease (49). Intriguingly, *Alistipes* has been found to decrease in responders and increase in nonresponders after an exercise intervention aimed to improve glucose homeostasis and insulin sensitivity in men with prediabetes (46). Further research on this genus in the context of PA is needed to understand its health effects, its ability to detect nonresponders to exercise for different health outcomes, and its potential to improve response when decreased using strategies such as dietary modification.

A limitation of the current report includes the cross-sectional nature of the analyses, which precludes the ability to determine causality. In addition, potentially informative variables such as transit time were not measured. There was also a lack of diversity in the current cohort with respect to BMI. Only individuals with BMI ≥25 kg·m^−2^ were enrolled in the study, and the average BMI of the participants used for the current analysis was >30 kg·m^−2^. This may have selected for a population with a smaller range of daily PA in terms of higher PA intensity. The limited range of daily PA intensity may have impeded our ability to detect statistically significant associations with or differences in fecal microbiota composition based on PA intensity. This may also have contributed to differences in the current findings relative to those reported in the exercise literature, which often involve athletes or structured exercise interventions. Common findings of these studies include increases in *Bifidobacterium*, *Lactobacillus*, and *Akkermansia* as well as butyrate and butyrate-producing taxa in response to exercise (19). More intense or vigorous exercise may be needed to induce these specific changes in the gut microbiota of sedentary individuals, as well as those with overweight or obesity (50). As mentioned previously, exercise is a subcategory of PA but is distinct in that it only includes planned, structured PA and is done with the objective of improving or maintaining physical fitness (2). Although this type of PA is beneficial for human health and physical fitness, evidence also suggests that small amounts of exercise may not be able to overcome the detrimental effects of a sedentary lifestyle and that increased daily PA (steps, standing, etc.) may be just as important for metabolic health, cancer, and mortality risk (51,52). Thus, it is important to distinguish between these two aspects of PA as they may have distinct effects on the gut microbiota and health in different contexts. Because of these limitations, the current analysis was considered exploratory, and therefore, *P* values were not adjusted. Thus, there is a higher likelihood of false discoveries, and results should be considered preliminary.

Intervention studies are needed to assess connections between the fecal microbiota and PA in a broader range of individuals, as well as with a broader range of potential modifying factors, such as medication use and transit time (53), to better elucidate the effects of PA on the gut microbiota as well as determine whether aspects of PA such as type, intensity, frequency, or duration influence the effect on the gut microbiota. This research may elucidate some of the mechanisms by which the gut microbiota mediates the health benefits of PA. Current research suggests that these mechanisms may include improved gut barrier function, insulin sensitivity, and mental health as well as reduced inflammation (20). Furthermore, emerging research on the gut–muscle and gut–bone axes indicates that the gut microbiota may contribute to the effects of exercise on muscle hypertrophy and bone health (20). In addition, future research on the topic of interindividual variability in response to daily PA should investigate the potential effect of differences in the gut microbiota. This could lead to gut-targeted dietary recommendations or probiotic supplements that could complement daily PA to enhance or enable beneficial metabolic responses in previously nonresponsive or less responsive individuals.

## CONCLUSIONS

In summary, the current analysis provides novel insights into the relationship between objectively measured PA and the gut microbiota composition of individuals with overweight and obesity that suggest potential beneficial or protective effects of PA on the gut microbiota, such as higher abundance of *Faecalibacterium* and lower abundance of *Alistipes*. This work provides a reference and foundation for future research on this topic.

## Supplementary Material
