# Supplementary material for: Associations between Accelerometer-Measured Physical Activity and Fecal Microbiota in Adults with Overweight and Obesity
Source: Med Sci Sports Exerc. 2022 Dec 22;55(4):680–9. doi: 10.1249/MSS.0000000000003096 (PMC9997628; doi:10.1249/MSS.0000000000003096)

**Supplementary Figure 1.** **Differences in SCFA profiles based on meeting PA recommendations**. Isovalerate and the SCFA/BCFA ratio were significantly different between groups that met and did not meet weekly PA recommendations. Abbreviations: branched chain fatty acids (BCFA), short-chain fatty acids (SCFA).


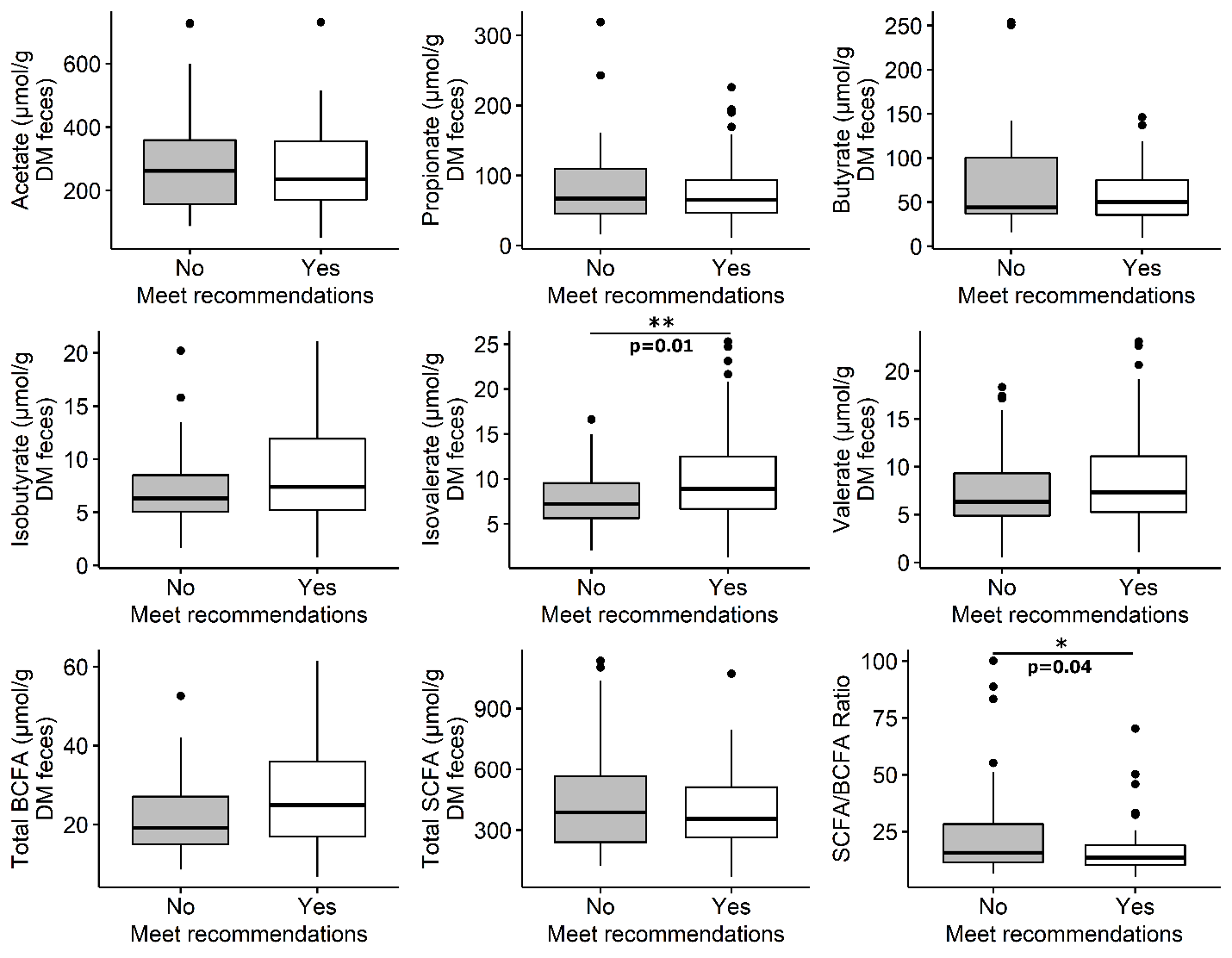


**Supplementary Figure 2.** **Meet PA cluster DESeq2 comparison**. DESeq2 identified three taxa, *Ruminococcus* (family Lachnospiraceae), YS2, and *Haemophilus*, that were significantly different between groups that met or did not meet weekly PA recommendations. Relative abundance of taxa displayed as proportions.


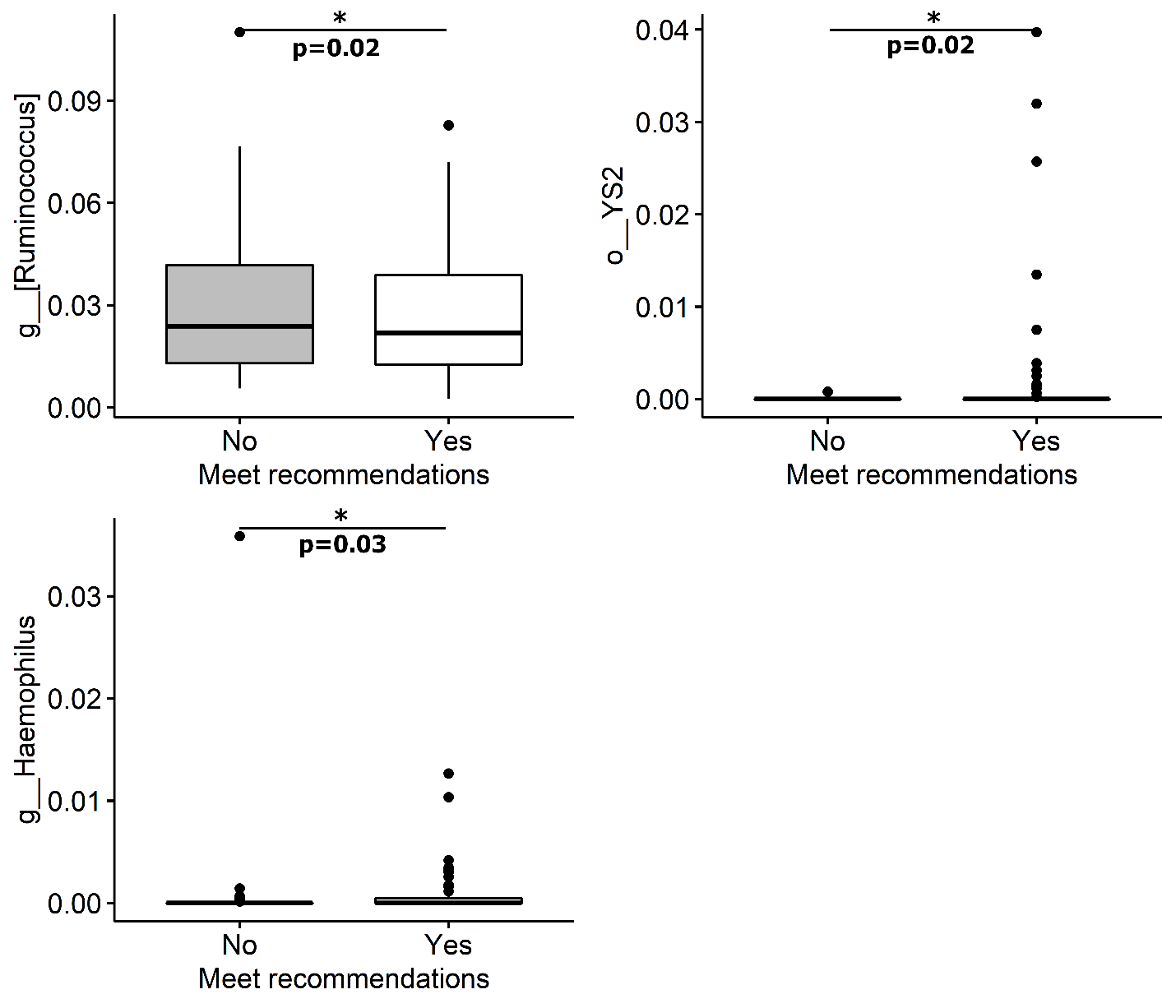


**Supplementary Figure 3.** **K-means cluster PA comparison**. Clusters showed significant differences in percent of time spent in MVPA and ST.


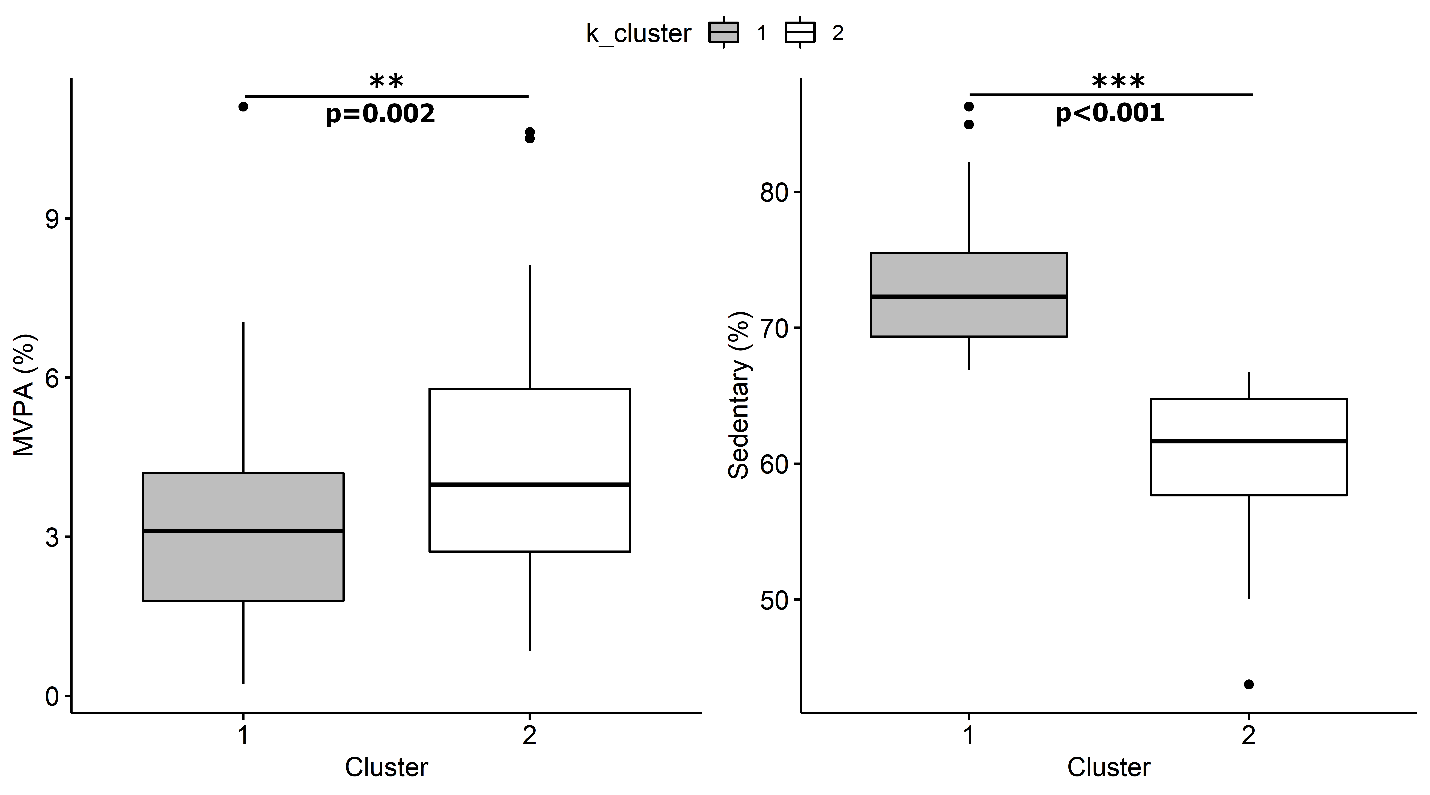

Supplement: Supplementary file 9 [file msse-55-680-s009.docx]
